# Arp2/3 complex and β1 integrin drive an invasive front through extracellular matrix adaptation in pancreatic cancer

**DOI:** 10.1002/ijc.70376

**Published:** 2026-03-07

**Authors:** Xiufen Yang, Yina Qiao, Yifeng Sun, Tamer Abdelaal, Kathleen Schuck, Hend Abdelrasoul, Carolina De La Torre, Malte Hermes, Yan Dong, Jingxiong Hu, Chao Fang, Xiaoyan Huang, Christoph Kahlert, Ingrid Herr, Christoph W. Michalski, Bo Kong

**Affiliations:** ^1^ Department of General, Visceral and Transplantation Surgery University of Heidelberg Heidelberg Germany; ^2^ Department of General and Visceral Surgery Ulm University Hospital Ulm Germany; ^3^ Orthopaedic and Sports Medicine Center, Beijing Tsinghua Changgung Hospital School of Clinical Medicine, Tsinghua University Beijing China; ^4^ Core Facility Platform Mannheim, NGS Core Facility, Medical Faculty Mannheim Heidelberg University Mannheim Germany

**Keywords:** Arp2/3, extracellular matrix, integrin, pancreatic cancer, type I collagen

## Abstract

Pancreatic ductal adenocarcinoma (PDAC) is one of the deadliest malignancies, due to its aggressive invasiveness and resistance to therapy. The dense, stiff extracellular matrix, composed primarily of collagen I and basement membrane components such as collagen IV and laminin, acts as a mechanical barrier that constrains PDAC invasion. We investigated whether the actin‐related protein (Arp) 2/3 complex, a key actin nucleator, is essential for PDAC cells to overcome extracellular matrix stiffness and facilitate migration. CRISPR/Cas9 knockout of the *Arpc4* gene in murine PDAC cell lines derived from Kras^G12D^‐driven transgenic mice resulted in substantially downregulated all Arp2/3 complex members. Inactivation of Arp2/3 significantly impaired PDAC cell migration, disrupted branched tubular structure formation in collagen I, and inhibited invasive front formation in organoid culture together with tumor‐associated macrophages and fibroblasts. Mechanistically, β1 integrin signaling emerged as a key regulator of Arp2/3‐dependent migration through collagen‐rich matrices. Clinically, elevated expression of Arp2/3 complex components correlates with poor patient survival and basal‐like differentiation subtypes, underscoring its role in disease progression. This study identifies the Arp2/3 complex and β1 integrin signaling as critical mediators of PDAC invasiveness and suggests them as potential therapeutic targets for mitigating PDAC progression.

## Graphical abstract

What's new?

Pancreatic ductal adenocarcinoma is characterized by a dense extracellular matrix, and how invasive tumor cells overcome such biomechanical constraints remains unclear. This study identifies the actin‐related protein 2/3 complex as a key regulator of pancreatic ductal adenocarcinoma invasiveness, driving fibroblast migration through β1 integrin signaling. In an organoid culture, loss of the Arp2/3 complex impaired cell migration, disrupted invasive front formation, and altered tumor–stroma interactions. Clinically, elevated Arp2/3 complex expression correlated with poor prognosis and basal‐like pancreatic ductal adenocarcinoma. These findings highlight the Arp2/3 complex as a potential therapeutic target to limit tumor progression in aggressive pancreatic ductal adenocarcinoma subtypes.

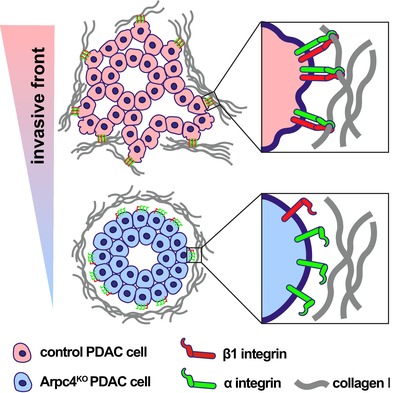

**Abbreviations**
ADM: acinar‐to‐ductal metaplasia
Arp2/3 complex: actin‐related protein 2/3 complex
BMDMs: bone marrow–derived macrophages
CAFs: cancer‐associated fibroblasts
ECM: extracellular matrix
PDAC: pancreatic ductal adenocarcinoma
PSCs: pancreatic stellate cells
RNAseq: RNAsequencing
scRNAseq: single‐cell RNAsequencing

## 1 INTRODUCTION

Pancreatic ductal adenocarcinoma (PDAC) is characterized by an excessive accumulation of extracellular matrix (ECM) proteins, a phenomenon known as desmoplastic reaction. This dense stroma is a defining feature of PDAC and contributes significantly to disease progression and therapy resistance. Specific desmoplastic stromal signatures have been shown to predict patient outcomes.^1^ The ECM plays a paradoxical role in PDAC, functioning both as a promoter and a restraint of tumor progression.^2^ Collagens, the predominant ECM components, regulate tumor growth, survival, and therapy resistance by modulating the biochemical and biomechanical properties of the stroma.^2^, ^3^, ^4^ However, while certain collagen subtypes promote oncogenesis, excessive collagen deposition has been paradoxically linked to improved patient prognoses, likely reflecting distinct roles of collagen subtypes.^5^, ^6^

Notably, type I collagen forms a stiff fibrotic matrix, which serves as a mechanical barrier that can suppress PDAC invasiveness by inducing stiffness‐dependent mechanosignaling.^7^, ^8^ Additionally, other ECM components, such as collagen IV and laminin, are key constituents of the basement membrane. This specialized ECM layer serves as a physical and biochemical barrier, restricting the transition of pre‐neoplastic cells into an invasive phenotype. Understanding how PDAC cells overcome the physical constraints of the dense ECM matrix is essential for understanding tumor invasion mechanisms and identifying actionable therapeutic targets.

The actin‐related protein (Arp) 2/3 complex consists of the subunits Arp2, Arp3, and Arpc1‐Arpc5.^9^ These subunits work together as a central actin nucleator. Unique in its function, the Arp2/3 complex binds to pre‐existing actin filaments and nucleates new branches, forming a dense actin network critical for cell motility and morphological adaption.^10^ Activation of the Arp2/3 complex occurs downstream of Rac1 (Rac family small GTPase 1) signaling in response to external stimuli, facilitating plasma membrane protrusions and actin stress fiber formation.^11^ This actin remodeling process is crucial for generating biomechanical forces required for cell migration and the formation of cellular structures like lamellipodia and filopodia.^11^

Previously, using an in vivo model of PDAC, we demonstrated the critical role of the Arp2/3 complex in early pancreatic carcinogenesis.^12^, ^13^ Specifically, Arp2/3‐mediated actin‐nucleation facilitated the formation of a basolateral actin cortex, which is essential for acinar‐to‐ductal metaplasia (ADM), a key adaptation of acinar cells in response to inflammatory cues. More recently, we reported that the Arp2/3 complex is required for cancer‐associated fibroblasts (CAFs) to adopt a myofibroblastic phenotype and facilitate efficient migration.^14^

Here, we focus on the role of the Arp2/3 complex in facilitating PDAC migration and morphological adaptation within stiff type I collagen and basement membrane matrices.

## 2 MATERIALS AND METHODS

### 2.1 Cell lines and culture conditions

The primary mouse PDAC cell lines 8025 and R254 were kindly provided by Dr. Dieter Saur and Dr. Günter Schneider (Department of Gastroenterology, Technical University of Munich, Munich, Germany). The 8025 cell line was derived from primary cells isolated from genetically engineered *p48* ^*Cre/+*^; *LSL‐Kras* ^*G12D/+*^ (*KC*) mice, while the R254 cell line originated from *p48* ^*Cre/+*^; *LSL‐Kras* ^*G12D/+*^; *p53* ^*flox/flox*^ (*KPC*) mice. Both cell lines were cultured in Dulbecco's modified Eagle medium (DMEM, Gibco, Waltham, MA) supplemented with 10% fetal bovine serum (FBS, Gibco, Waltham, MA), 100 U/mL penicillin and 100 μg/mL streptomycin (Merck, Darmstadt, Germany). Cultures were maintained at 37°C in a humidified incubator with 5% CO₂.

### 2.2 CRISPR/Cas9‐mediated gene editing of murine PDAC cell lines

The primary, murine PDAC cell lines 8025 and R254 were transfected with the CRISPR/Cas9 p20‐ARC Double Nickase Plasmid (Santa Cruz Biotechnology, Dallas, TX) to target the *Arpc4* gene, along with a negative control plasmid (Santa Cruz Biotechnology, Dallas, TX). Transfections were performed using Lipofectamine 3000 reagent (Thermo Fisher Scientific, Waltham, MA) according to the manufacturer's instructions. After 48 h of transfection, single cells with green fluorescence were sorted via fluorescence‐activated cell sorting (FACS) and subjected to selection with puromycin (InvivoGen, Toulouse, France) at concentrations of 0.7 mg/mL for 8025 cells and 1.2 mg/mL for R254 cells. Following 5–7 days of incubation under puromycin selection, the cells were seeded into 96‐well plates for clonal expansion. Clones with *Arpc4* knockout (*Arpc4* ^*ko*^) were confirmed by Western blot analysis.

### 2.3 Fluorescence‐activated cell sorting

Green fluorescent 8025 and R254 single cells were sorted via fluorescence‐activated cell sorting (FACS). Compensation was calculated in BD FACSDiva v6.1.3 using single‐stained controls (one control per fluorochrome). PMT voltages were first set on the unstained control to place negatives in the first decade; the FACSDiva Compensation Setup routine then generated the spillover matrix, which was applied during acquisition. The matrix was verified by re‐acquiring the single‐stained controls to confirm minimal residual signal in off‐target channels. Fluorescence‐activated cell sorting (FACS) was performed on a BD platform running FACSDiva v6.1.3. Single‐cell suspensions were stained with Dead Cell Stain Kit (L10119, Thermo Fisher Scientific, Germany). Acquisition and sorting were controlled by the use of FACSDiva v6.1.3, with compensation from single‐stained controls applied at acquisition. Events were gated sequentially: (i) stable acquisition period, (ii) FSC/SSC to exclude debris, (iii) doublet discrimination (FSC‐W vs. FSC‐H; SSC‐W vs. SSC‐H), (iv) live cells (viability: APC‐Cy7‐negative), (v) marker‐defined subsets (GFP positive) for sorting. Sort fractions were collected into protein‐supplemented medium on ice. A quality control after sorting included immediate re‐acquisition to confirm purity and viability.

### 2.4 Western blot analysis

Control and *Arpc4* knockout 8025 and R254 cells were lysed in ice‐cold RIPA buffer supplemented with protease and phosphatase inhibitors (Roche, Mannheim, Germany). Protein concentrations were measured using the BCA protein assay kit (Pierce, Thermo Scientific, Waltham, MA). Equal amounts of 20 μg protein were separated on 8–15% polyacrylamide gels and transferred to PVDF membranes (Roche, Mannheim, Germany). The membranes were blocked with 5% bovine serum albumin, then incubated with primary antibodies overnight at 4°C. After three washes in TBST buffer, the membranes were incubated with secondary antibodies for 1 h at room temperature, washed again, and visualized by chemiluminescence. Primary rabbit antibodies were: anti‐Arp2 (#AP3861), anti‐Arp3 (#AP4581), and anti‐Arpc1b (#AP4321), all from ECM Biosciences (Versailles, KY); anti‐Arpc4 pAb (#ab217065, abcam, Cambridge, UK); anti‐Hsp90 (#4877), anti‐MLC2 (#3672), and anti‐p‐MLC2 (Ser19, #3671) were from Cell Signaling Technology, Frankfurt, Germany. The primary mouse anti‐β1‐integrin antibody (#MA5‐17103) was from Thermo Fisher Scientific (Waltham, MA). The secondary antibodies were Donkey HRP‐labeled anti‐rabbit IgG (#NA‐934, GE Healthcare, Buckinghamshire, UK); Sheep HRP‐labeled anti‐mouse IgG (#NA‐931, GE Healthcare, Buckinghamshire, UK); IRDye® 680RD Goat anti‐Rabbit IgG (#926‐68071, LI‐COR, Bad Homburg, Germany).

### 2.5 Proliferation assay

Cells at 70%–80% confluence were harvested and counted. A total of 2000 cells (8025) or 1000 cells (R254) were seeded evenly into each well of a 96‐well plate. Five replicate wells were prepared for each experimental group. Following incubation for 24, 48, 72, or 96 h at 37°C with 5% CO_2_, 10 μL of CCK‐8 reagent (MedChemExpress, Monmouth Junction, NJ) was added to each well. The plates were then incubated for an additional 2 hours under the same conditions. Optical density (OD) values were measured at a wavelength of 450 nm using a microplate reader. Data were normalized to the baseline measurement at timepoint 0 and subjected to statistical analysis following previously published methods.^15^

### 2.6 Wound healing assay

This assay was performed by seeding 2 × 10^6^ cells into each well of a 6‐well plate to establish a monolayer. A straight scratch was created using the tip of a plastic 100 μL pipette, and the wound area was monitored using an inverted microscope at 0, 12, and 24 h. Images were captured, and the wound closure rate was quantified using ImageJ (NIH, Bethesda, MA).^16^

### 2.7 ECM substrate‐dependent random cell migration assays

For these assays, 35 mm cell culture dishes were prepared by coating with the following Corning® substrates: 8 μg/cm^2^ collagen I, 3 μg/cm^2^ collagen IV, 8 μg/cm^2^ laminin (all from Merk, Darmstadt, Germany), and 3 μg/cm^2^ fibronectin (R&D Systems, Abingdon, UK) following the manufacturer's instructions. The coating solutions were applied to the dishes and incubated overnight at 4°C. Before usage, the solutions were discarded, and the dishes were washed with PBS. Additionally, 500 μL Matrigel® was used to coat some dishes, incubated at 37°C in a 5% CO₂ atmosphere for 20 min for solidification. Others were coated with 1 mL 0.1% gelatin (Sigma‐Aldrich, Darmstadt, Germany) following the same incubation conditions, with excess solution removed afterwards.

Cell suspensions, containing 5 × 10^4^ murine, primary PDAC control or *Arpc4* ^*ko*^ cells in 2 mL DMEM supplemented with 10% FBS, 100 U/mL penicillin, and 100 μg/mL streptomycin, were carefully added to the coated dishes. The cultures were then incubated at 37°C in a 5% CO₂ atmosphere for 12 h. For those groups receiving blocking antibody treatments, the media also included 100 μg/mL of IgG (I‐1195, Leinco Technologies, St. Louis, MO) or β1‐integrin antibody (AIIB2, Developmental Studies Hybridoma Bank, Iowa City, IA). Cell migration was monitored using a HF 2000 incubation chamber (Pecon, Ulm, Germany) and an Axio Observer Z1 bright‐field microscope (Zeiss, Göttingen, Germany). Images were captured at 15‐min intervals over 24 h, with data analysis performed using ImageJ (NIH, Bethesda, MA).^16^

### 2.8 Isolation of pancreatic stellate cells and macrophages

Quiescent pancreatic stellate cells (PSCs) were isolated from mice following a published protocol.^17^ Pancreata from C57BL/6J wild‐type mice were minced and enzymatically dissociated in GBSS containing 0.05% collagenase P for 10 min at 37°C. The digest was passed through a 100‐μm nylon mesh and washed in GBSS supplemented with 3% BSA. After centrifugation, the pellet was resuspended in 4.75 mL GBSS/3% BSA containing 28% Histodenz, and 3 mL of 3% BSA‐GBSS was gently overlaid. Following centrifugation at 1400*g* (without brake), the diffuse band immediately above the Histodenz–GBSS interface was collected, washed in PBS, and plated. PSCs were maintained in DMEM supplemented with 5% FBS, 1% L‐glutamine, and 1% penicillin–streptomycin at 37°C in 5% CO₂, and used within 2 weeks. Bone marrow–derived macrophages (BMDMs) were generated from mice following established procedures.^18^ Briefly, tibiae and femora were aseptically excised and placed in PBS. After removal of surrounding soft tissues, the bone ends were trimmed and the marrow was flushed into DMEM (~5 mL per flush, repeated until the bones appeared white). Cell suspensions were pelleted (1500 rpm, 8 min) and supernatants discarded. Erythrocytes were lysed, cells were resuspended in PBS, passed through 100‐μm strainers into 50‐mL tubes, washed, and centrifuged again. The final pellet was resuspended to the desired density in DMEM supplemented with 20% FBS and M‐CSF (20 ng/mL), plated, and differentiated for 7 days before downstream functional analyses.

### 2.9 Organoid formation

Organoids were generated within collagen I (Sigma‐Aldrich, Darmstadt, Germany) using a 24‐well plate, with a total gel mixture volume of 400 μL. To prepare the gel, 13 μL neutralizing solution (550 mM HEPES in PBS) was added to 257 μL PDAC control or *Arpc4* ^*ko*^ cell suspension (500 cells/mL), followed by 130 μL of type I collagen. After thorough mixing, the gel mixture was transferred into a 24‐well plate and incubated at 37°C with 5% CO_2_ for 15 min to solidify. Subsequently, 600 μL DMEM containing 10% FBS, 100 U/mL penicillin, and 100 μg/mL streptomycin was added. The gel was detached from the well by encircling it with a 10 μL pipette tip and cultivated at 37°C with 5% CO_2_ for 10 days. The medium was refreshed every 2–3 days, and images were captured to document growth.

For organoids formed in Matrigel® (Corning, Berlin, Germany), 200 murine, primary PDAC control or *Arpc4* ^*ko*^ cells were suspended in 50 μL Matrigel®. The mixture was carefully added to a well of a 24‐well plate to form a dome shape and incubated at 37°C with 5% CO_2_ for 15 min to solidify. Following solidification, 500 μL organoid full medium^19^ was added, and the organoids were cultivated at 37°C with 5% CO_2_ for 10 days. The medium was refreshed every 2–3 days, and images were taken to record growth.

### 2.10 Organoid culture

The day prior, organoids in Matrigel® derived from the murine, primary PDAC cell line 8025 were prepared by collecting them using cold medium. These organoids were mechanically dissociated into smaller fragments through gentle pipetting and further processed into single cells (from smaller organoids) using TrypLE™. They were then seeded into a 24‐well plate and cultured in full organoid medium for 24 h. On the following day, a unit solution was prepared containing 75% Matrigel®, 25% collagen I, 5 μg/mL fibronectin (R&D systems, UK), and 2.5 μg/mL vitronectin (R&D systems, UK). Additionally, mouse PSC primary cells (5.0 × 10^5^ cells) and bone marrow‐derived macrophages (10 × 10^5^ cells) were prepared as previously described.^18^, ^20^

The model consisted of two distinct layers. The first layer included a mixture of 187.5 μL Matrigel®, 62.5 μL collagen I, 5 μg/mL fibronectin, and 2.5 μg/mL vitronectin, which was added without cells and incubated at 37°C for 20 min. The second layer was comprised of 562.5 μL Matrigel®, 187.5 μL collagen I, 5 μg/mL fibronectin, and 2.5 μg/mL vitronectin, combined with 2.5 × 10^5^ organoids, 5 × 10^5^ PSC cells, and 10 × 10^5^ macrophages. This mixture was layered on top of the first layer and incubated at 37°C for 20 min. Subsequently, 1 mL of 5% FBS DMEM reduced medium was added, and the co‐culture was maintained for 5 days. Following incubation, the 3D co‐culture micro tumors were harvested for RNA‐sequencing (RNAseq) and H&E staining as previously described.^12^

### 2.11 RNA extraction and RNAseq

For total RNA extraction from organoids, TRIzol reagent (Thermo Fisher, Waltham, MA) was added to the gel after removing the supernatant. The gel was dissolved by thorough mixing. Chloroform (Thermo Fisher) was added, followed by vigorous vortexing for 15 s. After incubation at room temperature for 3 min, the mixture was centrifuged at 12,000*g* for 15 min at 4°C. The upper aqueous phase was carefully transferred to a fresh tube, and an equal volume of ≥95% ethanol was added. Total RNA was then purified using the Monarch Total RNA Miniprep Kit (New England Biolabs, Frankfurt am Main, Germany) according to the manufacturer's instructions. For 2D‐cultured cells, total RNA was directly extracted using the Monarch Total RNA Miniprep Kit (New England Biolabs) following the manufacturer's protocol.

RNAseq from organoid samples was conducted at the NGS Core Facility, Medical Faculty Mannheim, University of Heidelberg, Mannheim, Germany. RNAseq for 2D‐cultured samples was performed at the Core Unit Bioinformatics, Medical Faculty, University of Ulm, Germany. RNAseq for samples embedded in three different matrices was performed by Biomarker Technologies (BMKGENE, Münster, Germany). The sequencing coverage and quality statistics for each sample are summarized (Table S1).

Differential gene expression analysis was carried out using the Bioconductor software package DESeq2 (https://bioconductor.org/packages/release/bioc/html/DESeq2.html), with genes showing |Log2FC| > 1 and *p*‐value <0.05 considered differentially expressed. Pathways and Ontologies analysis were performed by the use of the online tool Enrichr (https://maayanlab.cloud/Enrichr/) with down regulated genes (Arpc4^KO^ versus control) from each RNAseq data.

### 2.12 Gene set variation analysis

The computational method Gene Set Variation Analysis (GSVA; https://www.gsea-msigdb.org/gsea/index.jsp) was applied to evaluate the contribution of the eight ARP complex genes (ARPC1A, ARPC1B, ARPC2, ARPC3, ARPC4, ARPC5, ACTR2, and ACTR3) to the overall variation in the TCGA‐PAAD dataset. GSVA‐based scores were computed for further analysis using GSVA version 1.47.0.

### 2.13 Clinical dataset acquisition

The study utilized data from The Cancer Genome Atlas (TCGA, https://www.cancer.gov/ccg/research/genome-sequencing/tcga), which offers comprehensive genomic and clinical information on PDAC patients. Expression profiles and associated clinical outcomes were extracted for analysis. Overall survival was assessed using Cox regression models and Kaplan–Meier survival curves. Following previously published work,^21^, ^22^ samples were dichotomized into high‐risk and low‐risk groups using the maximally selected log‐rank statistic based on overall survival in the TCGA‐PAAD cohort (*n* = 177), yielding the cut‐off that maximized separation of the Kaplan–Meier curves. The correlation analysis between the ARP complex and basal‐like or classical subtypes was performed using the Pearson method.

### 2.14 Single‐cell RNAseq data analysis

Single‐cell RNAseq (scRNAseq) datasets were obtained from GSE155698 in the Gene Expression Omnibus (GEO). Data preprocessing and quality control were conducted following previously described methods.^12^, ^14^ Copy number variations (CNVs) were calculated to identify malignant tumor cells using the “copycat” R packages. Cell clusters were identified using the Seurat package's FindClusters function, applying a graph‐based clustering approach with a resolution range of 0.05–0.25. Candidate gene expression was visualized using dot plots generated with the dotplot function.

### 2.15 Statistical analysis

All analyses were performed using R version 4.0.4 (https://www.bioconductor.org/) and GraphPad Prism version 7 (https://www.graphpad.com/). Statistical comparisons for categorical and numerical variables were carried out using Mann–Whitney tests, while correlations were evaluated using Pearson's correlation. The Cox proportional hazards regression model was implemented using the R “survival” package. Single‐cell data analysis was conducted with the “Seurat” package version 4.0.1, and dot plots were created using a combination of the “Seurat” and “ggplot2” packages. A two‐tailed Student's *t* test was used to assess the significance of differences between group means, with a *p*‐value <0.05 considered statistically significant. Results are expressed as mean ± standard error.

## 3 RESULTS

### 3.1 Arp2/3 complex inactivation impairs PDAC migration on type I collagen and basement membrane matrices

To elucidate the functional significance of the Arp2/3 complex in PDAC cell migration, we employed CRISPR/Cas9 to knock out the *Arpc4* gene in murine PDAC cell lines derived from Kras^G12D^‐driven transgenic mouse models (8025 and R254).

**FIGURE 1:**
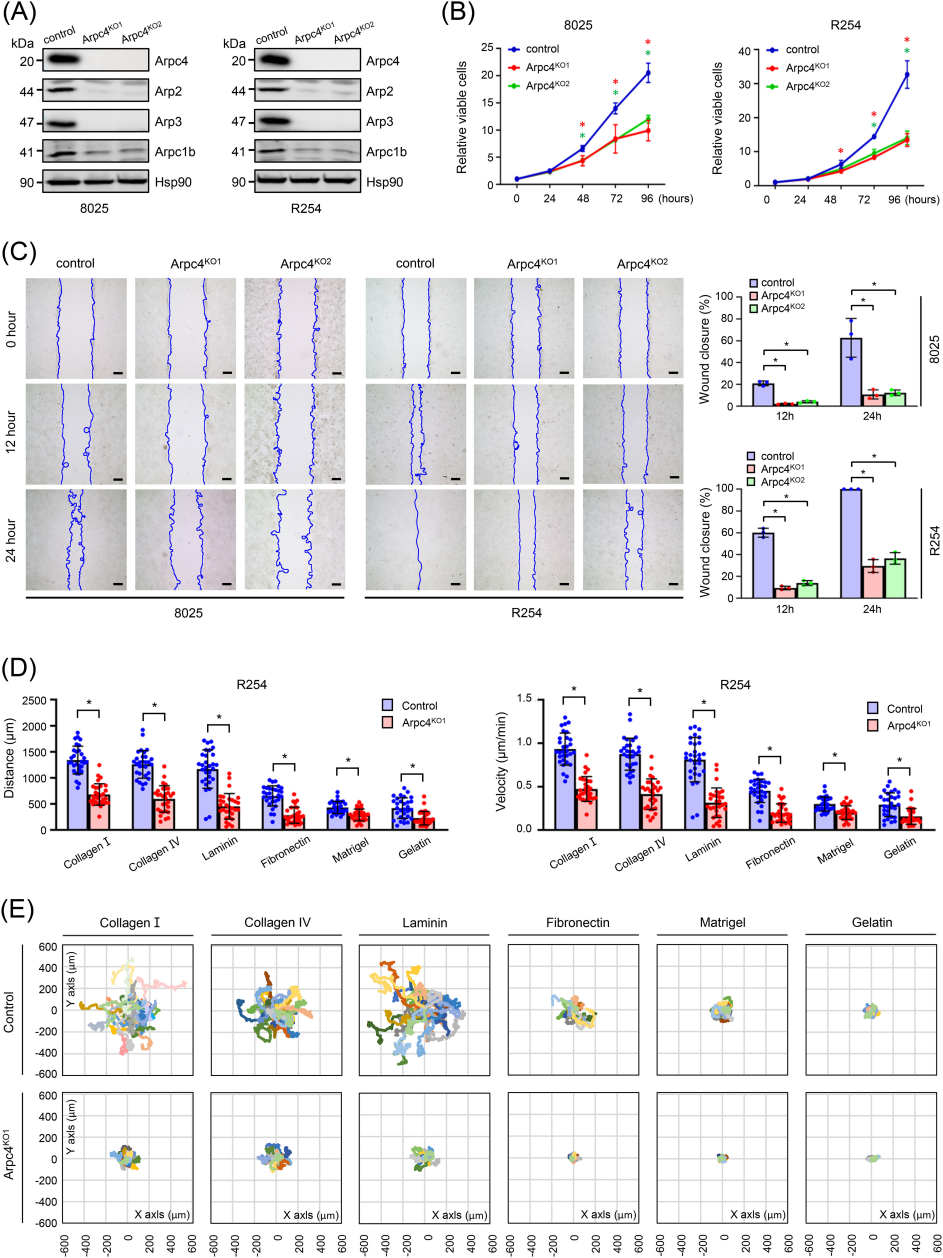
Arp2/3 complex inactivation reduces PDAC migration on type I collagen and basement membrane matrix. (A) Western blot analysis shows the expression levels of Arp2/3 complex subunits Arpc4, Arp2, Arp3, and Arpc1b in 8025 and R254 cells under control and Arpc4 knockout conditions. (B) The proliferation assay was conducted on 8025 or R254 control and Arpc4 knockout cells. The cell viability of cells in each group was assessed using a CCK‐8 assay on the specified hours (0, 24, 48, 72, 96), with data (OD_450_ values) normalized to the measurement at time point 0. *p < 0.05, unpaired *t* test. For 8025 cells, *p*‐values for Arpc4^KO1^ versus control are 0.0221 (48 h), 0.0254 (72 h), and 0.0021 (96 h); for Arpc4^KO2^ versus control, they are 0.0046 (48 h), 0.0007 (72 h), and 0.0015 (96 h). For R254 cells, *p*‐values for Arpc4^KO1^ versus control are 0.0356 (48 h), <0.0001 (72 h), and 0.0017 (96 h); for Arpc4^KO2^ versus control, they are 0.0022 (72 h), and 0.0020 (96 h). (C) Representative images are shown to demonstrate the migration of cells into the wounded area at the indicated time points (scale bar: 200 μm). The percentage of wound closure was quantified and plotted to assess the cell migration rate. For 8025 cells, *p*‐values for Arpc4^KO1^ versus control are 0.0001 (12 h) and 0.0079 (24 h); for Arpc4^KO2^ versus control, they are 0.0003 (12 h) and 0.0083 (24 h). For R254 cells, *p*‐values for Arpc4^KO1^ and Arpc4^KO2^ versus control are <0.0001 at both 12 h and 24 h. (D) Migrated distance (left) and velocity (right) of R254 cells on plates coated with various ECM components (type I collagen, type IV collagen, laminin, fibronectin, Matrigel® and gelatin) are shown in the charts. *p < 0.0001, unpaired *t* test. (E) Panels depict single‐cell trajectories on plates coated with type I collagen, type IV collagen, laminin, fibronectin, Matrigel® and gelatin. Thirty randomly selected cell trajectories are shown for each group.

Western blot analysis confirmed a complete loss of Arpc4 expression, accompanied by a substantial reduction in Arp2, Arp3, and Arpc1b levels (Figure 1A). These findings suggest a near‐total disruption of Arp2/3 complex functionality.

Functionally, *Arpc4*‐depletion significantly impaired cell proliferation and migration in both 8025 and R254 PDAC cells (Figure 1B,C). To quantitatively assess migration, we performed random cell migration assays using time‐lapse video microscopy on plastic surfaces pre‐coated with various ECM proteins: collagen I, collagen IV, laminin, fibronectin, gelatin, and Matrigel®, as previously described.^23^

Control PDAC cells migrated significantly faster on collagen I, collagen IV, and laminin compared to fibronectin, gelatin, and Matrigel® (Figure 1D). In contrast, *Arpc4*‐deficient PDAC cells exhibited markedly reduced migration across all tested ECM substrates. Additionally, these cells displayed more constrained migration trajectories, particularly on collagen I, collagen IV, and laminin. This effect was less pronounced on gelatin, fibronectin, and Matrigel® (Figure 1E). These results highlight the critical role of the Arp2/3 complex in facilitating effective PDAC cell migration, especially in collagen‐rich and basement membrane‐like matrices.

### 3.2 Arp2/3 complex inactivation impairs morphological adaptation in collagen I and invasive front formation in mixed ECMs

**FIGURE 2:**
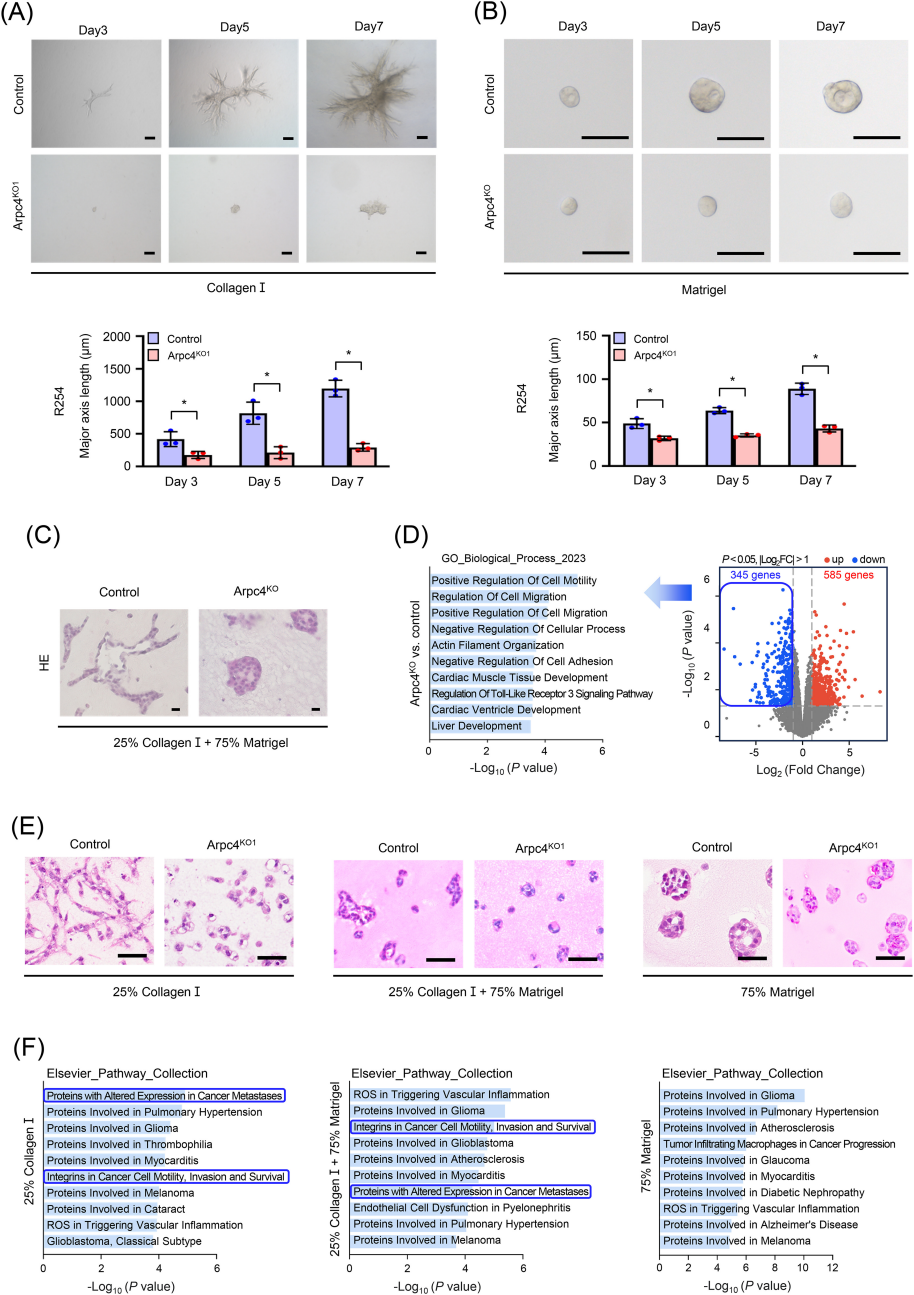
Arp2/3 inactivation impairs morphological adaptation in type I collagen and formation of an invasive front in mixed ECMs. (A) Representative phase‐contrast pictures show the morphology of control and Arpc4^KO^ R254 cells in type I collagen (left, scale bars: 100 μm) at various time points. Quantitative analysis of size for the structures formed by PDAC cells (right). *p*‐values for Arpc4^KO^ vs. control are 0.0298 (Day 3), 0.0056 (Day 5) and 0.0004 (Day 7). (B) Representative phase‐contrast pictures show the morphology of control and Arpc4^KO^ R254 cells in Matrigel® (left, scale bars: 100 μm) at various time points. Quantitative analysis of size for the structures formed by PDAC cells (right). *p*‐values for Arpc4^KO^ vs. control are 0.0070 (Day 3), 0.0002 (Day 5), and 0.0005 (Day 7). (C) Representative H&E‐stained sections display the morphology of the structure formed in 3D co‐culture matrix by control and Aprc4^KO^ 8025 cells, scale bars: 20 μm. (D) Volcano plot of RNAseq results comparing Arpc4^KO^ versus control in 8025 cell‐derived 3D structures (right). Functional annotation of downregulated genes (*n* = 345) after Arpc4 knock‐out in murine PDAC 8025 cells (left), and top 10 annotations are shown. Reference genome: GRCm39. (E) Representative H&E‐stained sections display the morphology of the structure formed in 25% type I collagen, 25% type I collagen + 75% Matrigel® and 75% Matrigel® by control and Aprc4^KO^ 8025 cells on the 5th day, scale bars: 50 μm. (F) Functional annotation of downregulated genes after Arpc4 knock‐out of RNAseq results comparing Arpc4^KO^ versus control of 8025 cells in 25% type I collagen, 25% type I collagen + 75% Matrigel® and 75% Matrigel®. Top 10 annotations are shown. Reference genome: GRCm39.

To investigate the morphological responses of murine PDAC cells in different extracellular matrices, we analyzed their behavior in two key matrix environments: rigid collagen I and the less rigid Matrigel®, which contains basement membrane components such as collagen IV and laminin. The reduced rigidity of Matrigel®, compared to collagen I, offers a contrasting environment for cellular dynamics.^24^ We embedded single control and Arp knockout (*Arpc4* ^*KO*^) cells into floating collagen I gels or Matrigel®, and tracked their migration and morphological changes over a period of 7 days. In the rigid collagen I matrices, control PDAC cells formed branched tubular structures during migration, a process markedly impaired in cells with the Arp2/3 complex inactivated (Figure 2A). Conversely, in Matrigel®, cells predominantly formed spheroids instead of branched structures, with the spheroids growing in diameter, as observed with bright‐field microscopy after 7 days (Figure 2B). The growth of these spheroids was partially compromised by the inactivation of the Arp2/3 complex.

Given the absence of detectable type I collagen in Matrigel®,^3^ we further enhanced our experimental system by supplementing Matrigel® with collagen I. Additionally, to simulate the key cellular components of the PDAC microenvironment, we included naive PSCs and bone marrow‐derived macrophages to represent and tumor‐associated macrophages (TAMs), respectively, creating an ex vivo microtumor model. Notably, in this co‐culture system, control cells developed gland‐forming spheroid bodies with multiple invasive fronts, surrounded by dense stroma and closely resembling the histological architecture of PDAC in vivo. In contrast, *Arpc4* ^*KO*^ cells exhibited compromised invasive front formation, resulting in more solid, less invasive tumors (Figure 2C).

To elucidate the mechanisms underlying these observations, we performed RNAseq on microtumors derived from control and *Arpc4* ^*KO*^ cells and consisting of TAMs, PSCs, and organoids. Differential gene expression analysis identified 345 genes significantly downregulated in *Arpc4* ^*KO*^ tumors compared to controls (*p* < 0.05, Log2FC: <−1). Pathway and ontology analysis indicated that “positive regulation of cell motility” and “positive regulation of cell migration” were the most significantly enriched terms (Figure 2D), emphasizing the critical role of cell migration within the collagen I matrix in driving observed morphological differences. To rule out the impact of CAFs and TAMs on PDAC migration, we performed a migration assay using control and *Arpc4* ^*KO*^ cells on plates coated with a matrix of 75% Matrigel® and 25% collagen I, in the absence of CAFs and TAMs. Here, *Arpc4* ^*KO*^ cells showed consistently reduced migration speeds and more confined trajectories compared with control cells (Figure S1A,B).

To isolate matrix effects independent of TAMs and CAFs, we repeated the experiment without TME cells and embedded control and *Arpc4* ^*KO*^ cells in three matrices: 25% collagen I, 25% collagen I + 75% Matrigel®, and 75% Matrigel® for 5 days, followed by H&E staining and RNAseq. Control organoids showed a clear matrix‐dependent trend: branched tubular structures in 25% collagen I, gland‐forming spheroids with invasive fronts in 25% collagen I + 75% Matrigel®, and compact spheroids in 75% Matrigel®. In contrast, *Arpc4* ^*KO*^ organoids remained largely spheroidal across all conditions, with only minimal formation of tubular structure in 25% collagen I (Figure 2E). Transcriptomically, 495, 578, and 611 genes were downregulated in *Arpc4* ^*KO*^ versus control cells in 25% collagen I, 25% collagen I + 75% Matrigel®, and 75% Matrigel®, respectively (Figure S2A–C). Pathway analysis of downregulated genes identified “Integrins in Cancer Cell Motility, Invasion and Survival” as the top enriched term in 25% collagen I and 25% collagen I + 75% Matrigel®, but not in 75% Matrigel® (Figure 2F). These data suggest that collagen I promotes an integrin‐dependent PDAC motility program associated with tubular morphology and invasive fronts.

### 3.3 β1 integrin signaling facilitates Arp2/3‐dependent migration and morphological adaptation in collagen I matrix

**FIGURE 3:**
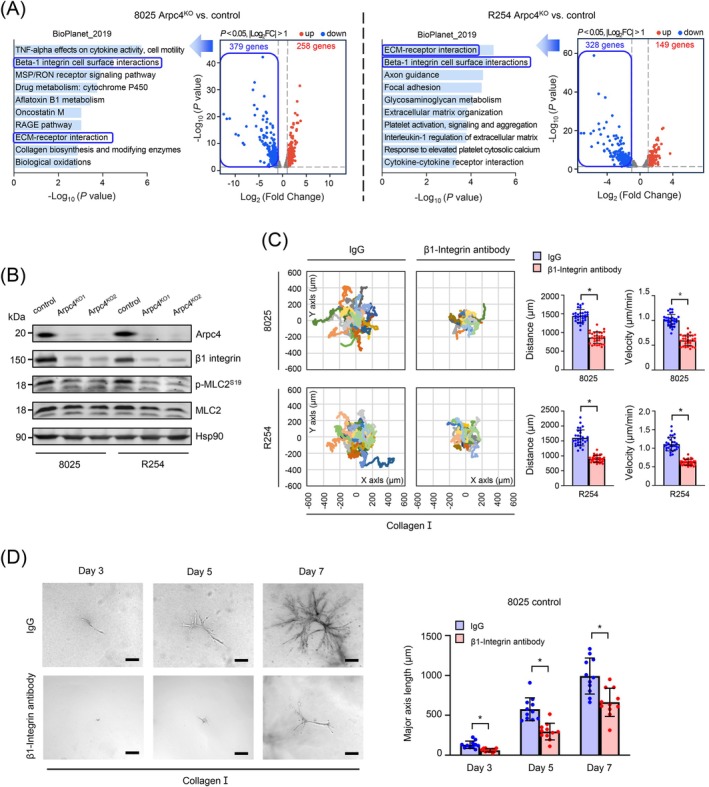
β1 integrin signaling facilitates Arp2/3‐dependent migration on type I collagen. (A) Volcano plot of RNAseq results comparing Arpc4^KO^ versus control in 8025 and R254 cells. Functional annotation of downregulated genes (8025 cells, *n* = 379; R254 cells, *n* = 328) after Arpc4 knock‐out in murine PDAC cells. Top 10 annotations are shown. Reference genome: GRCm39. (B) Western blot shows expression levels of Arpc4, β1 integrin and p‐MLC2 in control and Arpc4^KO^ cells, Hsp90 as a loading control. (C) Panels depict single‐cell trajectories on plates coated with type I collagen with treatment of 100 μg/mL IgG or β1‐Integrin antibody. Quantitative analysis for migrated distance and velocity of cells in each group. (D) Representative phase‐contrast pictures show the morphology of control 8025 cells in type I collagen (left, scale bars: 200 μm) with treatment of 100 μg/mL IgG or β1‐Integrin antibody at various time points. Quantitative analysis of size for the structures formed by PDAC cells (right). *p*‐values for β1‐Integrin antibody vs. IgG are 0.0002 (Day 3), <0.0001 (Day 5), and 0.011 (Day 7).

To explore the intrinsic role of the Arp2/3 complex in cell migration within PDAC, we grew the two murine PDAC cell lines, 8025 and R254, in 2D on plastic substrate, followed by RNAseq to compare *Arpc4* ^*KO*^ and control cells across. This analysis revealed 379 and 328 genes significantly downregulated in *Arpc4* ^*KO*^ cells in the 8025 and R254 lines, respectively. Functional annotation of these genes emphasized the importance of “β1 integrin cell surface interactions” and “ECM‐receptor interactions” as the top enriched pathways in both cell lines (Figure 3A). Western blot analysis supported these findings, showing reduced β1 integrin protein levels in *Arpc4* ^*KO*^ cells compared to controls. Additionally, levels of phosphorylated Myosin Light Chain 2 (Ser19, p‐MLC2) were also decreased, indicating a reduction in biomechanical force generation essential for effective migration (Figure 3B). To directly assess the impact of β1 integrin signaling on cell migration, we conducted migration assays on collagen I‐coated plates using either a control IgG or a β1 integrin‐blocking antibody. The results demonstrated that PDAC cells treated with the β1 integrin‐blocking antibody showed significantly reduced migration speeds and more restricted migration trajectories on collagen I compared to those treated with control antibodies (Figure 3C); however, no such effect was observed on Matrigel® or Laminin matrices (Figure S3). Finally, we embedded single PDAC cells in floating 3D collagen‐I gels and tracked morphology for 7 days in the presence of either a control IgG or a β1‐integrin‐blocking antibody. Inhibition of β1‐integrin disrupted the formation of branched tubular structures relative to control, phenocopying loss of Arp2/3 function (Figure 3D). These results indicate that β1‐integrin signaling also supports Arp2/3‐dependent branched tubular morphogenesis in collagen I matrix.

### 3.4 Increased ARP2/3 complex expression correlates with poor prognosis and basal‐like subtype in PDAC

**FIGURE 4:**
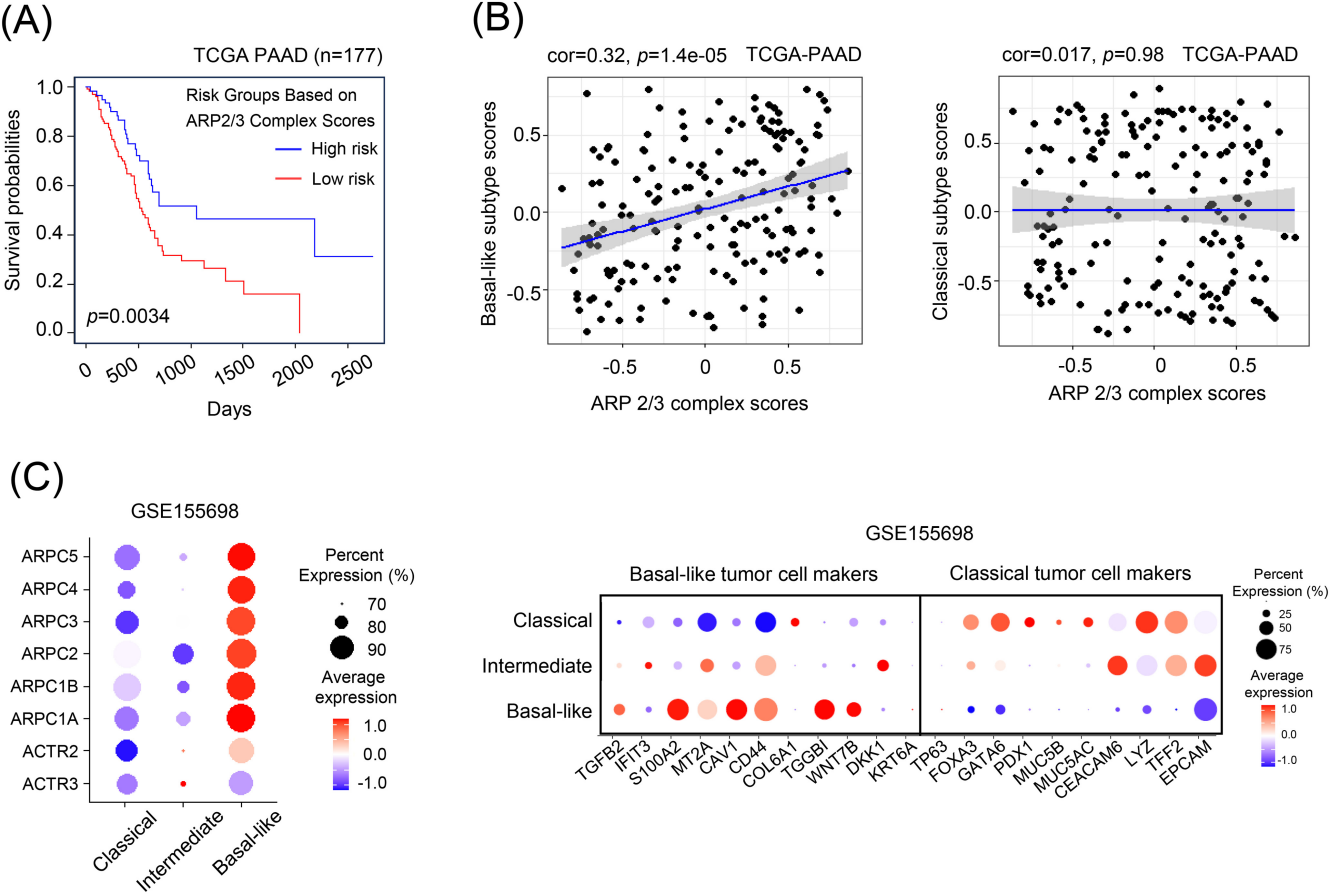
Increased expression of ARP2/3 signatures correlates with poor prognosis and basal‐like subtype in PDAC. (A) Kaplan–Meier survival curve for PDAC patients of TCGA datasets‐“TCGA Pancreatic Adenocarcinoma” (TCGA‐PAAD) project, with high‐ and low risk‐GSVA‐based scores for ARP2/3 complex signature (N^high‐risk^ = 115 vs. N^low‐risk^ = 62; median survival: 470 vs. 627 days, log‐rank test: *p* < 0.01). (B) Pearson correlation analysis between basal‐like subtype‐specific or classical type‐specific scores and ARP2/3 complex expression. (C) Dot plot demonstrates the expression of ARP2/3 complex subunits and representative markers in classical, basal‐like and intermediate state of PDAC cell from human PDACs samples (GSE155698).

In our clinical translation study, we utilized a Gene Set Variation Analysis (GSVA) to develop a scoring system for each ARP2/3 complex transcript based on expression levels from the TCGA dataset (*n* = 177).^25^ Our analysis revealed that PDAC patients with high‐risk ARP2/3 complex scores demonstrated shorter overall survival compared to those with low‐risk scores (Figure 4A). We then explored whether the ARP2/3 complex signature was particularly enriched in any preexisting molecular subtypes of PDAC. Using established molecular classifiers, we correlated the ARP2/3 complex risk scores with molecular subtypes. According to Moffitt's classification,^26^ the risk score of the ARP2/3 complex was significantly associated with the basal‐like subtype, but not with the classical subtype (Figure 4B). Furthermore, leveraging single‐cell RNA sequencing (scRNAseq) data from human PDAC samples,^27^ we conducted quality control and copy number variation (CNV) correction before identifying three clusters with representative marker expressions for basal‐like, classical, and intermediate states (*n* = 4020 cells).^28^ Significantly higher expression levels of ARP2/3 complex subunits were observed in the basal‐like cluster compared to the classical and intermediate states (Figure 4C).

## 4 DISCUSSION

Our findings establish the Arp2/3 complex as a pivotal cellular mechanism enabling PDAC cells to navigate through stiff collagen I matrices, overcoming their potential tumor‐restraining mechanical properties. This function is characterized by significant morphological adaptations, including altered migration trajectories and the formation of an invasive front within mixed ECM environments. Additionally, we identified β1‐integrin as a crucial downstream mediator, facilitating ECM‐dependent migration and structural adaptations.

Our data are in line with previous studies that underscored the role of the Arp2/3 complex in enhancing PDAC migration and progression. For example, high ARP3 expression was associated with poor survival in PDAC patients, and its inhibition disrupted the migration capabilities of human PDAC cell lines.^29^ Likewise, ARPIN, an endogenous Arp2/3 complex inhibitor, was shown to be markedly reduced in liver metastases compared to primary tumors.^30^

We have expanded the already known knowledge by leveraging bioinformatics tools and recent omics datasets. By this way we demonstrated that elevated expression of the seven Arp2/3 complex subunits correlates with poor prognosis, particularly in the basal‐like subtype of PDAC. Correspondingly, recent data demonstrated that organoids with branched structures embedded in collagen gels more accurately reflect the phenotypic heterogeneity observed in human PDACs.^31^ It is conceivable that differences in the migration capacities of PDAC subclones within collagen I contribute to this heterogeneity. Our study augments these insights by providing functional evidence of the critical role of the Arp2/3 complex in developing these branched structures.

Notably, integrin pathways and the Arp2/3 complex frequently collaborate with the Arp2/3 complex in various biological contexts. For instance, while the Arp2/3 complex is not universally necessary for phagocytosis or chemotaxis in macrophages, it is vital for integrin‐based adhesion during motility on fibronectin‐coated substrates.^32^ During embryonic development, integrins form adhesive belts around Arp2/3 complex‐dependent actin protrusions, creating invadosome‐like structures.^33^ Consistent with these findings, we noted a significant reduction in β1‐integrin protein levels in Arpc4^KO^ PDAC cells, without a corresponding decrease in mRNA expression. This suggests that the Arp2/3 complex‐mediated actin network might regulate β1‐integrin endocytosis, trafficking, and recycling, as noted in prior studies.^34^, ^35^ Additionally, our observations indicate that Arp2/3 complex inactivation notably impairs PDAC migration on collagen I and IV, laminin, and fibronectin matrices, where PDAC cells depend on β1‐integrin for ECM interaction.^36^, ^37^ Further high‐quality biochemical studies are required to elucidate the precise molecular mechanisms of this interaction.

In conclusion, our study underscores the essential role of the Arp2/3 complex and β1‐integrin in the migration and morphological dynamics of PDAC cells within challenging ECM environments. We have demonstrated how the interplay between these molecules facilitates cell movement through mechanically restraining collagen matrices and supports invasive growth patterns, highlighting their significance in cancer progression and metastasis. These findings not only contribute to our understanding of the molecular mechanisms driving PDAC but also suggest potential therapeutic targets for disrupting tumor spread, particularly in aggressive basal‐like subtypes. Future studies are warranted to further dissect the detailed interactions between the Arp2/3 complex and integrin‐mediated pathways, potentially paving the way for the development of targeted treatments that could impair cancer cell migration and invasion, ultimately improving patient outcomes in pancreatic cancer.

## AUTHOR CONTRIBUTIONS

**Xiufen Yang:** Investigation. **Yina Qiao:** Investigation; writing – original draft. **Yifeng Sun:** Investigation. **Tamer Abdelaal:** Investigation. **Kathleen Schuck:** Investigation. **Hend Abdelrasoul:** Investigation. **Carolina De La Torre:** Investigation. **Malte Hermes:** Investigation. **Yan Dong:** Investigation. **Jingxiong Hu:** Investigation. **Chao Fang:** Investigation. **Xiaoyan Huang:** Investigation. **Christoph Kahlert:** Writing – review and editing. **Ingrid Herr:** Writing – original draft; writing – review and editing. **Christoph W. Michalski:** Writing – review and editing. **Bo Kong:** Conceptualization; methodology; validation; formal analysis; supervision; funding acquisition; project administration; writing – original draft.

## FUNDING INFORMATION

This study was supported by the Hans Beger Stiftung (No. 17‐02‐2022 to B.K.) and by the Wilhelm Sander‐Stiftung (No. 2022.135.1 to B.K.).

## CONFLICT OF INTEREST STATEMENT

The authors declare no conflicts of interest.

## ETHICS STATEMENT

The procurement of organs from deceased mice for the isolation of macrophages and pancreatic stellate cells received approval from the institution.

## Supporting information


**Table S1.** Provided as a separate Excel file.

**Figure S1.** Arp2/3 inactivation impairs migration of PDAC cells on mixed ECM. (A) Panels depict single‐cell trajectories of control and Aprc4KO 8025 cells on plates coated with 25% type I collagen + 75% Matrigel. Thirty randomly selected cell trajectories are shown for each group. (B) Migrated distance (left) and velocity (right) of 8025 cells on plates coated with 25% type I collagen + 75% Matrigel are shown in the charts. *p < 0.0001, unpaired *t*‐test.
**Figure S2.** RNA‐seq Volcano plot of PDAC cells in different matrices. Volcano plot of downregulated genes after Arpc4 knock‐out of RNA‐seq results comparing Arpc4KO versus control of 8025 cells in (A) 25% type I collagen, (B) 25% type I collagen + 75% Matrigel, and (C) 75% Matrigel.
**Figure S3.** Migration of PDAC cells with/without treatment of β1‐Integrin antibody on other matrices. Panels depict single‐cell trajectories of control 8025 cells on plates coated with (A) Matrigel or (B) Laminin with treatment of 100 μg/mL IgG or β1‐Integrin antibody. Quantitative analysis for migrated distance and velocity of cells in each group.

## Data Availability

Data sources and handling of the publicly available datasets used in this study are described in section 2. The sequencing data have been deposited at https://www.ncbi.nlm.nih.gov/geo/query/acc.cgi?acc=GSE284423 and https://www.ncbi.nlm.nih.gov/geo/query/acc.cgi?acc=GSE305670. Other data that support the findings of this study are available from the corresponding author upon request.
