# Supplementary material for: Arp2/3 complex and β1 integrin drive an invasive front through extracellular matrix adaptation in pancreatic cancer
Source: Int J Cancer. 2026 Mar 7;158(11):3038–50. doi: 10.1002/ijc.70376 (PMC13047250; doi:10.1002/ijc.70376)
Supplement: Supplementary file 2 — Figure S1. Arp2/3 inactivation impairs migration of PDAC cells on mixed ECM. (A) Panels depict single‐cell trajectories of control and Aprc4KO 8025 cells on plates coated with 25% type I collagen + 75% Matrigel. Thirty randomly selected cell trajectories are shown for each group. (B) Migrated distance (left) and velocity (right) of 8025 cells on plates coated with 25% type I collagen + 75% Matrigel are shown in the charts. *p < 0.0001, unpaired t‐test. Figure S2. RNA‐seq Volcano plot of PDAC cells in different matrices. Volcano plot of downregulated genes after Arpc4 knock‐out of RNA‐seq results comparing Arpc4KO versus control of 8025 cells in (A) 25% type I collagen, (B) 25% type I collagen + 75% Matrigel, and (C) 75% Matrigel. Figure S3. Migration of PDAC cells with/without treatment of β1‐Integrin antibody on other matrices. Panels depict single‐cell trajectories of control 8025 cells on plates coated with (A) Matrigel or (B) Laminin with treatment of 100 μg/mL IgG or β1‐Integrin antibody. Quantitative analysis for migrated distance and velocity of cells in each group. [file IJC-158-3038-s001.pdf]

Supplementary Materials for

Arp2/3 complex and β1 integrin drive an invasive front through extracellular matrix adaptation in pancreatic cancer

Xiufen Yang, Yina Qiao, Yifeng Sun, Tamer Abdelaal, Kathleen Schuck, Hend Abdelrasoul, Carolina De La Torre, Malte Hermes, Yan Dong, Jingxiong Hu, Chao Fang, Xiaoyan Huang, Christoph Kahlert, Ingrid Herr, Christoph W. Michalski and Bo Kong

This PDF file includes:

Figs. S1 to S3 with legends

Supplementary Table S1 is provided as a separate Excel file

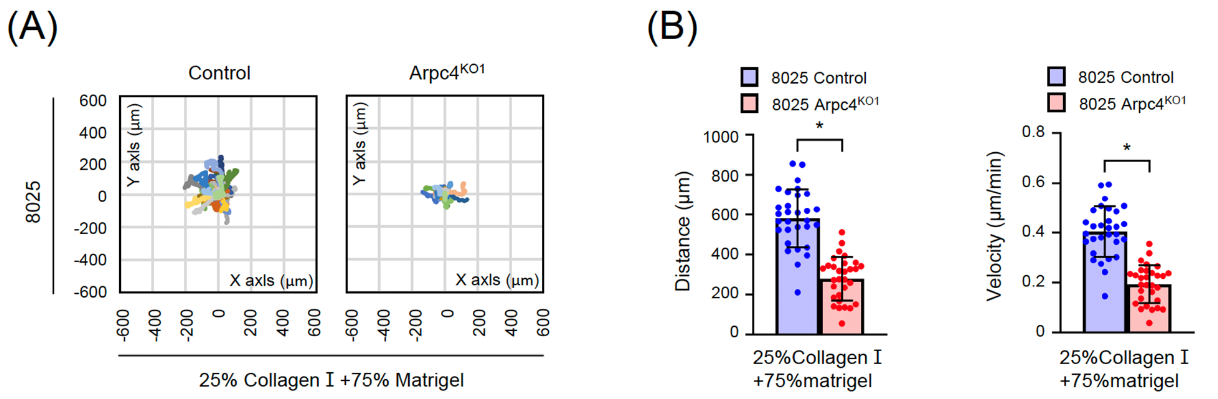

Fig. S1 Arp2/3 inactivation impairs migration of PDAC cells on mixed ECM. **(A)** Panels depict single-cell trajectories of control and Aprc4^{KO} 8025 cells on plates coated with 25% type I collagen + 75% Matrigel. Thirty randomly selected cell trajectories are shown for each group. **(B)** Migrated distance (left) and velocity (right) of 8025 cells on plates coated with 25% type I collagen + 75% Matrigel are shown in the charts. *p < 0.0001, unpaired t-test.

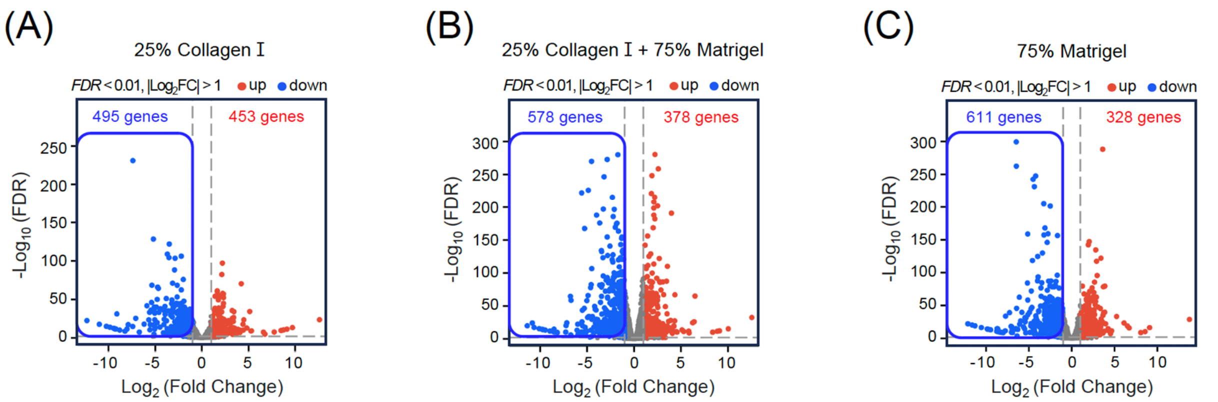

Fig. S2 RNA-seq Volcano plot of PDAC cells in different matrices. Volcano plot of downregulated genes after Arpc4 knock-out of RNA-seq results comparing Arpc4^{KO} versus control of 8025 cells in **(A)** 25% type I collagen, **(B)** 25% type I collagen + 75% Matrigel, and **(C)** 75% Matrigel.

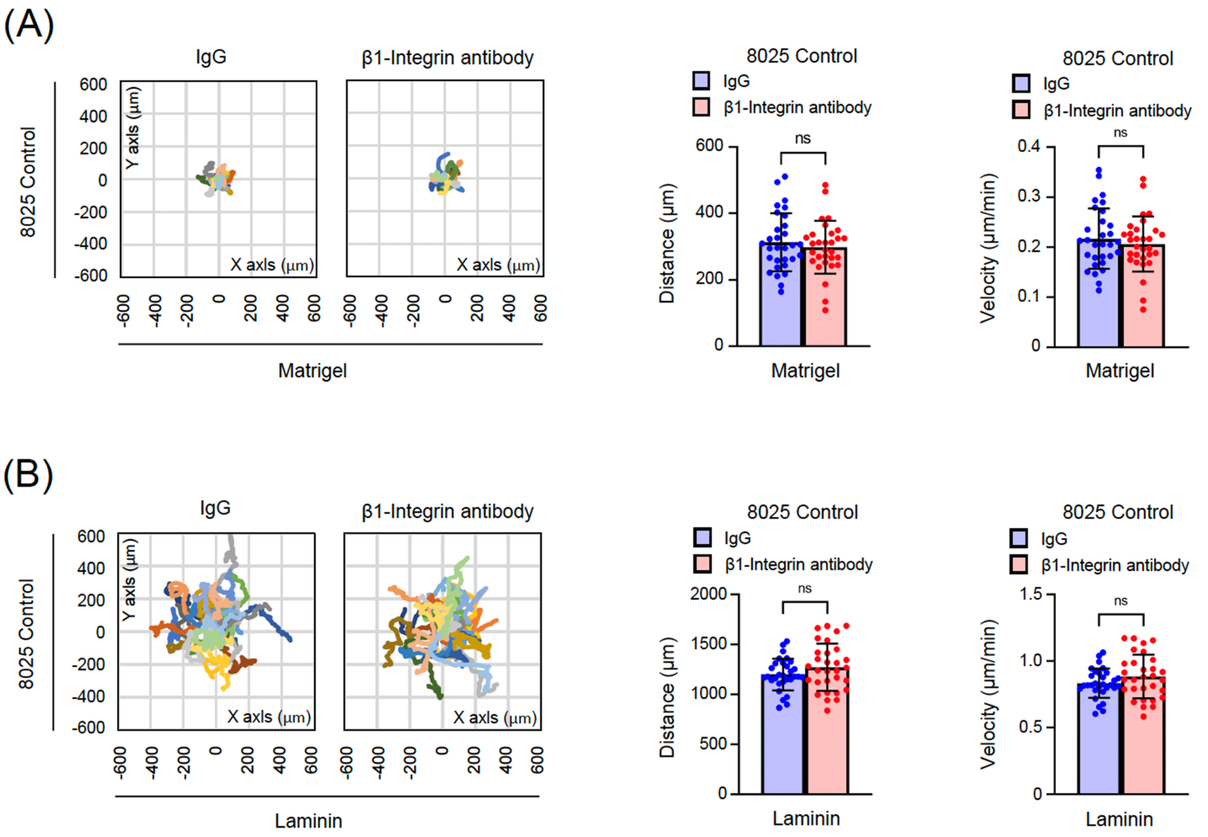

Fig. S3 Migration of PDAC cells with/without treatment of β1-Integrin antibody on other matrices. Panels depict single-cell trajectories of control 8025 cells on plates coated with (**A**) Matrigel or (**B**) Laminin with treatment of 100 μg/ml IgG or β1-Integrin antibody. Quantitative analysis for migrated distance and velocity of cells in each group.
